# Supplementary material for: Are carbon emissions trading and green financial instruments synergistic? -Comprehensive quantitative research based on content analysis
Source: PLoS One. 2024 Mar 7;19(3):e0298601. doi: 10.1371/journal.pone.0298601 (PMC10919601; doi:10.1371/journal.pone.0298601)
Supplement: S1 Data — (ZIP) [file pone.0298601.s001.zip › Data/地级市Z_F工作报告.zip!/╜¡╦╒/╗┤░▓╩╨/└┤╫╘╣1⁄2╓┌║┼í╛┬φ┐╦╩2╛▌═°í┐_2017.docx]

各位代表：

现在，我代表市人民政府向大会作工作报告，请予审议，并请各位政协委员和其他列席同志提出意见。

一、2016年工作回顾侯卫东官场笔记

一年来，市政府认真落实中央和省委、省政府的决策部署，按照市委的工作要求，紧紧围绕建设全面小康社会和苏北重要中心城市两大目标，自觉践行新发展理念，扎实推进供给侧结构性改革，统筹抓好改革发展稳定各项工作，经济社会保持平稳健康发展，实现了“十三五”发展的良好开局。

（一）经济运行稳中有进。实现地区生产总值3048亿元、同比增长9%，一般公共预算收入完成省政府指导性目标和市人大常委会调整后的任务，主要经济指标增幅保持在合理区间。各项投入较快增长。完成规模以上固定资产投资2535亿元、规模以上工业投资1518亿元，分别增长15.1%和17.3%。220个市级重大项目完成投资1040亿元、增长11.5%，14个列入部省试点PPP项目吸纳资金规模达68亿元，年末社会融资总额4178亿元、增长26%，持续加大的有效投入成为全市经济稳增长的“压舱石”。实体经济平稳发展。进一步放大联席会议、千企帮扶等六大平台功能，协调金融机构为企业发放低息贷款15亿元、转贷资金40亿元，努力为实体经济发展创造良好环境。全市工业完成入库税金170亿元、增长5%，企业实现利润367亿元、增长8%。建筑业加快转型发展，总产值突破1500亿元。全面启动“五证合一”登记改革，推行市场准入“双告知双承诺”制度，放宽市场主体注册条件，深入推进全民创业、淮商崛起行动，进一步激发了市场活力，新登记私营企业1.6万户、个体工商户4万余户。“五大任务”有效落实。累计化解水泥产能60万吨、钢铁产能137万吨，淘汰低端低效铁合金产能8万吨、纺织产能近1万吨、铅蓄电池产能100万千伏安时，依法处置地条钢企业9户，关停小化工企业26户。全市商品住房去化周期由2015年底的15.9个月减少到9个月。银行业机构不良贷款率降至1.82%，较年初下降0.56个百分点。贷款加权平均利率较年初下降0.39个百分点，新增减免涉企收费项目18个，一般工商业及其他用电每度降低3.12分，为小微企业减免税费近3亿元。制定落实28项补短板任务，发展的整体性、协调性和可持续性进一步增强。创新能力加快提升。扎实推进国家创新型示范城市建设，获批创建全国质量强市示范城市。全社会研发投入占GDP比重达1.8%，高新技术产业产值占比达25%。新增国家高新技术企业63户、企业“两站三中心”94个、省级企业科技服务站15个，新建校企联盟18家，企业专利申请量和授权量快速增长，有效发明专利万人拥有量达3.5件。引进国家“千人计划”人才21名、省“双创”人才20名。

（二）现代产业加快发展。先进制造业扩量提质。“4+2”优势特色产业实现产值4100亿元，盐化新材料、特钢及装备制造等产业保持较快增长，食品产业突破千亿元，新开工德科玛半导体、时代芯存等一批投资超百亿元重大项目，敏安电动汽车获批新能源汽车生产牌照，为产业发展提供了新的支撑。“两化融合”取得积极成效，完成工业技改投入650亿元，全市单位GDP能耗下降完成省定目标任务。特色服务业活力增强。实施投资超5000万元项目200个，完成投资603亿元、增长14.9%。29家省、市级现代服务业集聚区入驻企业超过1.3万户，营业收入增长13%。实现服务业增加值1455亿元、增长10.6%，其中“4+3”特色服务业增加值占比达76%。旅游业、快递业收入和电子商务交易额分别增长14.3%、67%和33%，西游记文化体验园等4个项目入选全国优选旅游项目，成为全国旅游业改革创新先行区。现代农业增创优势。实施投资超3000万元农业产业化项目130个，“4+1”农业项目占比达77%，单个龙头企业年销售突破50亿元。新增家庭农场871户、土地股份合作社131个，新拓展高效设施园艺面积8.1万亩，发展稻虾共作等综合种养基地10万亩，新增地理标志证明商标15件，一批国家和省级农业园区的示范带动效应显著提升。加大农业设施投入，新增高标准农田近40万亩，秸秆机械化还田近700万亩，农业综合机械化、粮食收储和气象服务现代化步伐不断加快。

（三）改革开放全面深化。重点领域改革取得突破。行政审批制度改革深处发力，健全权责清单动态管理制度，实行投资建设项目“四联合”审批，推行多部门在线数字化联合图审，启动国家“互联网+政务服务”信息惠民工程试点，实现政府便民服务热线多号合一。构建全过程市场主体监管模式，建立市县一体化公共资源交易平台，盱眙相对集中行政许可权改革试点通过省级评估。新型城镇化综合改革深入推进，建成市区“多规合一”空间信息技术平台，顺利完成“两区一县”行政区划调整，涟水、金湖、洪泽和盱眙马坝等新型城镇化综合试点有序推进，城镇投融资机制改革取得积极成果，新推出4项居住证公共服务事项。土地使用制度改革加快实施，洪泽建设用地“双减量”试点取得阶段性成果，淮安区获批全国国土资源节约集约模范区。新型农村改革体系加快构建，土地承包经营权确权登记颁证基本完成，农村产权流转交易市场覆盖所有乡镇，探索农民宅基地有偿退出试点，金湖成为全国农村承包土地经营权抵押贷款试点县。金融改革成效显著，新开业4家银行机构，省信用担保公司在淮设立分支机构，11户企业登陆多层次资本市场，组建金融控股有限公司，核准发行企业债券52亿元。全面开展不动产统一登记，顺利完成党政机关公车改革，统筹推进国企、物价、供销和科技等体制改革。开放合作水平不断提升。台资企业产业转移集聚服务示范区建设获得省政府专门政策支持，获批国家级海峡两岸青年就业创业示范点，连续五年获评台商投资“极力推荐城市”。深度对接国家和省重大发展战略，加快“东融西拓、南联北接”步伐，全域融入江苏沿海开发战略，淮河生态经济带纳入国家“十三五”规划纲要，宁淮一体化发展进程提速。新引进亿元以上内资项目200个，其中10亿元以上项目20个；新批总投资3000万美元以上外资项目59个，其中1亿美元以上项目10个。新增进出口实绩企业140户，新设立境外投资企业15户，新布局“一带一路”重要节点国家友好交流城市2个。园区平台提档升级。淮安经济技术开发区通过国家级生态工业园区验收，淮安综保区列入跨境电商试点，淮安高新区创建国家级即将获批，获准筹建省级清河经济开发区和苏淮高新区，淮安软件园创成省级互联网产业园和企企通示范园区，施河教学具产业园创成国家级火炬特色产业基地。

（四）城乡建设统筹推进。中心城市建设迈出新步伐。投入845亿元，实施846个重点项目。功能片区建设加快推进，高铁商务区核心区建设全面启动，金融中心一期主体结构封顶，获批建设白马湖国家湿地公园，周恩来故里景区、河下古镇、码头古镇、蒋坝古镇保护与开发有序推进，完成1.6万户、193万平方米棚户区改造任务。城市公共交通不断优化，正式运营现代有轨电车一期工程，启动实施市区内环高架工程，改造瓶颈路段27个，新改建停车泊位2200多个，完成市区公交体制改革，新购700辆新能源公交车。实施应急避难场所、海绵城市等项目，建成管线地理信息系统和电梯应急处置平台。重大基础设施建设取得新进展。连淮扬镇、徐宿淮盐铁路建设快速推进，宁淮铁路前期工作正式启动。235国道、503和348省道、淮河出海航道整治、淮安新港二期等工程顺利实施，淮安涟水机场启动二期扩建工程。完成洪泽湖大堤除险加固、淮河入江水道和分淮入沂整治等重点工程，淮河入海水道二期同步实施二级航道工程前期工作取得重大进展。城乡环境建设取得新成效。深入开展全国文明城市和联合国人居环境奖城市创建，实施139个“九整治”和542个“三规范”项目，整治老旧小区230个，拆除违法建设15.8万平方米。优化28个片区和109个镇村布局规划，10个重点中心镇基本建成“十个一”功能性项目，新改建农村公路500公里，推行农村环境“五位一体”管护，创建美丽乡村32个。全面完成饮水安全工程建设任务，新解决41.86万农村人口饮水安全问题。生态文明建设取得新突破。出台实施《淮安市永久性绿地保护条例》和《淮安市古淮河保护条例》，国家生态市、国家节水型城市创建通过考核验收。全力打好空气污染防治攻坚战，PM2.5平均浓度同比下降8.4%，优良天数较上年增加3天、达248天，重污染天数减少4天。推行水环境区域补偿工作，县级以上集中式饮用水源地水质全面达标，8个地表水国考断面均值达到年度考核要求。

（五）民生保障持续加强。投入55.6亿元，全面完成十件民生实事项目，城乡居民人均可支配收入分别达30335元、14319元，分别增长7.9%和9.1%。公共服务能力不断增强。创建教育现代化先进学校60所、先进乡镇10个，高考二本以上录取数连续八年超过万人，新扩建省优质幼儿园23所，建成一批省级现代化职业学校、专业群和实训基地。综合医改深入推进，实施分级诊疗制度，推广家庭医生签约服务模式，创新总额控制下的病种结算办法，建成省级临床重点专科15个，平稳实施全面两孩政策。公共文化设施覆盖率达95%，版权登记总量大幅提升，淮安大剧院投入运营，恩来干部学院核心区主体工程竣工，周恩来纪念地景区入选“全国我最向往的党史纪念地”。成功举办国际智力运动精英赛、市第八届运动会，省级公共体育服务体系示范区实现全覆盖。社会保障体系日益健全。加大创业就业扶持力度，发放创业贷款1.3亿元，“零就业”家庭全部当月清零，城镇登记失业率为1.87%。实施精准扶贫，12.9万低收入农村人口脱贫，45个经济薄弱村达“新八有”标准，黄花塘革命老区获得省单独实施“点穴式”帮扶。城乡低保标准分别从每人每月490元、370元提高至520元、400元，健全社会救助和大病保险制度，建立多层次养老服务体系，落实困难残疾人生活补贴、重度残疾人护理补贴，做好困难群体精神关爱工作，实施机关事业单位工作人员养老保险制度改革。健全住房保障体系，发放住房公积金贷款32亿元。社会治理创新深入推进。圆满完成“六五”普法。探索警务体制机制改革，创新流动人口居住管理，刑事发案率明显下降。加强人民调解和社区矫正工作，实现城乡社区网格化社会服务管理全覆盖，建成透明放心菜市场和透明餐饮系统，积极打造阳光信访升级版，安全生产形势持续平稳向好。其他工作取得新进展。国防动员、双拥共建活动扎实开展，军民融合深度发展，实现全国双拥模范城“四连冠”，创成全国人防先进城市。对口支援工作富有成效，援疆项目建设进展顺利。完成第十一届村民委员会和第六届居民委员会换届选举工作。广播电视、机关事务、仲裁、档案、地方志、民族宗教、防震减灾、老龄、妇女儿童、青少年、关心下一代、慈善、红十字等事业加快发展。

（六）行政效能不断提升。坚持高点谋划。围绕落实“十三五”规划，深入谋划推进打基础、利长远的重大事项，出台实施优化空间功能定位和产业布局意见、融入沿海开发实施方案、台资示范区建设行动方案等政策文件，增强工作的前瞻性和主动性。及时制定供给侧结构性改革系列意见，实施助推实体经济发展、稳定外贸增长等创新举措，努力营造有利于企业发展和经济增长的大环境。坚持实处发力。面对淮安目前所处的发展阶段，牢固确立新型工业化第一方略工作导向，聚力培育产业、服务企业、招引项目，进一步增强工业经济的主体地位和支撑作用。坚持问题导向，强化组织协调，着力解决资金、用地、人才、房屋征收等方面的难题。进一步精简会议文件，大力推行101%服务，持续深化“两为”整治，限期解决招商引资中未兑现的承诺事项，努力营造重服务、敢担当、讲诚信的干事氛围。坚持严字当头。深入开展“两学一做”学习教育，认真落实从严治党、从严治政新要求。出台法治政府建设实施纲要，推进行政复议应诉和行政执法规范化建设，普遍建立政府法律顾问制度，政务公开、网络问政不断深化。完善行政决策程序，规范管理市级政府投资项目、政府债务和产业引导资金。认真贯彻中央八项规定和国务院“约法三章”精神，严格落实党风廉政建设主体责任和“一岗双责”，坚决查纠“四风”，从严控制“三公”经费支出，加大审计、监察力度，大力惩治各类腐败问题，着力营造风清气正的政务环境。

各位代表，2016年的各项成绩是在应对严峻挑战、克服重重困难的情况下取得的，既为实现“十三五”目标奠定了良好基础，也为本届政府圆满履职画上了句号。五年来，坚持以科学跨越发展为主线，全面小康建设取得阶段性成果。初步构建现代产业发展支撑体系，综合实力迈上新台阶，人均GDP超过全国平均水平，以县为单位达到省定2003版全面小康标准。对照高标准全面小康指标体系，加大攻坚力度，着力补齐短板，为高水平建成全面小康社会打牢了基础。五年来，坚持以特色城镇化为引领，苏北重要中心城市建设迈上新台阶。秉持“全域规划、多规合一”理念，初步构建了布局合理、层级分明、功能清晰、特色鲜明的新型城镇体系。优化了中心城市“双心三轴九组团”布局，建成区面积达180平方公里、人口增至160万人。县城承载能力显著提升，一批重点中心镇、特色镇初具规模，城镇化率达59.5%，实现了常住人口止跌回升的重大转变。五年来，坚持以改革开放为动力，科学跨越发展新动能日益增强。26项改革纳入国家和省级试点，引进实施投资超10亿元和超1亿美元项目140个，本土企业实现在A股主板上市，一批企业登陆多层次资本市场。打造了台资集聚示范区等一批国家级开放平台，建成了淮安智慧产业谷等一批创新创业载体，引进了科技领军、行业带头等一批高层次人才，创新逐步成为淮安发展的主引擎。五年来，坚持以重大基础设施为支撑，综合交通枢纽地位基本确立。两条高铁相继开工，机场一类航空口岸正式开放，盐河航道整治工程全面完成，多层次、高密度的公路网加快构建，立体式综合交通体系为淮安融入大战略、实施大开放拓展了新空间。五年来，坚持以保障改善民生为根本，社会和谐基础不断巩固。扎实推进民生幸福六大体系建设，促进公共服务资源向基层延伸、向农村覆盖、向弱势群体倾斜，城乡基本社会保险覆盖面达95%以上，各类民生保障标准稳步提高，教育、卫生、文化、体育事业协调发展，生态环境持续改善，发展成果更多更好地惠及人民群众。

各位代表，以上成绩的取得，是省委、省政府和市委坚强领导的结果，是市人大、市政协和社会各界有效监督、大力支持的结果，是全市人民团结拼搏、扎实苦干的结果。我谨代表市人民政府，向广大干部群众，向人大代表、政协委员和离退休老同志，向各民主党派、工商联、无党派人士和各人民团体，向驻淮部队、武警官兵和部省驻淮单位，向来淮投资创业者，向所有关心和支持淮安发展的海内外朋友，表示衷心的感谢并致以崇高的敬意！

五年的拼搏奋进，成绩来之不易，经验弥足珍贵。我们深深体会到，只有坚决贯彻中央大政方针和省市委决策部署，积极适应和引领经济发展新常态，才能把握大势、争取主动；只有勇于担当、敢于创新，才能抢抓机遇、破解难题；只有心无旁骛谋发展、扑下身子抓落实，才能打牢基础、厚植优势；只有着眼大局、维护团结，才能凝聚民心、汇聚力量；只有牢记宗旨、不忘初心，才能永葆本色、行稳致远。在看到成绩的同时，我们也清醒地认识到存在的问题和不足。面对经济发展新常态，破解难题手段还不多，自主创新能力还不强，产业支撑还不够有力，稳增长的基础还不够牢固。面对城乡一体化发展的新趋势，中心城市的集聚辐射能力与其定位还不相匹配，县域经济综合实力相对较弱，城乡发展差距仍然较大。面对群众过上美好生活的热切期盼，在基本公共服务均衡配置、打造宜居生态家园等方面还有明显短板，在加强社会治理创新、维护公共安全等方面还面临许多新矛盾。面对政府职能转变的新要求，服务效能有待提升，依法行政能力需要提高，求真务实作风还要进一步锤炼。这些发展中的问题，需要依靠加快发展予以解决。我们将牢记使命、砥砺奋进，不断开创各项工作的新局面。

二、今后五年的目标思路和2017年工作任务

未来五年，全国将实现第一个“百年目标”，全省将高水平全面建成小康社会，淮安迎来了又一个重要的发展机遇期，新一届政府肩负着全面建成小康社会和奠定苏北重要中心城市地位的历史重任。省第十三次党代会和市第七次党代会深刻分析了今后五年面临的机遇和挑战，进一步确立“两聚一高”新要求、深化“两大目标”新内涵，为淮安加快发展提供重要遵循、指明具体路径。市政府将认真贯彻落实中央和省市委的决策部署，进一步抢抓机遇、破解难题、增创优势，积极探索依托生态优势走出发展新路子，全面落实“十三五”规划确定的各项目标任务、战略举措和工作重点，确保全市经济运行稳中有进、稳中趋好，改革开放活力更强、空间更广，群众生活稳步提高、更加幸福，城乡建设统筹推进、协调发展，生态环境宜业宜居、优势凸显，社会治理体系完善、和谐稳定，努力实现更高质量、更有效率、更加公平、更可持续的发展，加快将淮安建设成为繁荣之城、安康之城、宜居之城、文明之城。

各位代表，今年是新一届政府履职的开局之年。市政府将全面贯彻党的十八大、十八届三中四中五中六中全会和习近平总书记系列重要讲话精神，认真落实中央、省经济工作会议和省、市党代会、市委七届三次全会部署，紧紧围绕“五位一体”总体布局和“四个全面”战略布局，坚持稳中求进总基调，以践行新发展理念为引领，以提高发展质量和效益为中心，以推进供给侧结构性改革为主线，紧扣“两聚一高”，聚焦“两大目标”，深入实施“六大战略”，更加突出“四个导向”，统筹抓好稳增长、促改革、调结构、惠民生、优生态、防风险各项工作，努力以优异的成绩喜迎党的十九大。今年全市经济社会发展主要预期目标是：地区生产总值增长8.5%，规模以上工业增加值增长9%，规模以上固定资产投资增长12%，一般公共预算收入同口径实现增长，社会消费品零售总额增长11%左右，城乡居民人均可支配收入分别增长7.5%左右和8.5%左右，居民消费价格涨幅控制在3%以内，城镇登记失业率控制在4%以内，节能减排确保完成省定任务。围绕上述目标，着重抓好七个方面工作：

（一）提升发展质效，加快新型工业化步伐。

夯实产业发展基础。突出新型工业化第一方略和工业强市主旋律，进一步聚集资源、强化保障，加大产业培育力度。重抓有效投入，加快实施省市重大项目，实现规模以上工业投资增长13%。重抓产业提升，突出产业横向连接和纵向延伸，加快发展新能源汽车等先进制造业，积极培育“专精特新”企业和产品，着力打造更多细分行业“单打冠军”，推进产业转型升级。重抓企业培育，实施骨干企业培育计划，促进成长型企业发展壮大，重点培植60户“4+2”产业龙头企业，新增开票销售超亿元企业30户，形成年销售2000万元以上新增长点200个。重抓全民创业，加大创业培训和扶持力度，加快推进工商登记全程电子化，培育更多市场主体。

增强自主创新能力。坚持企业主体地位，加快资金、人才、政策等创新要素向优质企业集聚，推动企业大力开展产品创新、管理创新和商业模式创新等创新活动，新增国家高新技术企业100户，力争高新技术产业产值占比达27%。主攻关键环节，聚焦盐化新材料、凹土资源开发等产业，实施重大装备技术和产品质量攻关项目，加快突破关键核心技术，力争在行业标准制定上掌握话语权。激活创新要素，推动政产学研协同创新，力争市级以上企业“两站三中心”达900个；强化人才支撑，新引进省“双创”人才项目15个、“淮上英才计划”项目60个。优化创新生态，进一步完善支持创新的体制机制，不断提高创新的便利性、对创新监管的宽松性和对创新人才的包容性，大力扶持科技孵化基地成长和高端人才创业。强化知识产权保护，创建“零侵权”城市。

优化企业发展环境。加大帮扶力度，帮助企业有效解决发展中的突出问题，不断激发企业发展活力。加大金融支持力度，逐步提高制造业贷款占比，企业直接债务融资余额超过600亿元，股权融资增加5亿元以上。加快建立产品研发、信息咨询和中介服务等公共平台，满足企业多样化需求。推进国家质量强市示范城市建设，新培育1-2户市长质量奖企业，新增著名商标15个、知名商标35个、省级名牌产品5-6个。盘活低效工业用地，拓展产业发展空间，提高土地产出率。

（二）转变发展方式，促进现代农业提质增效。

增强支撑保障能力。深入推进农业供给侧结构性改革，进一步优化供给结构和资源配置，提高农业综合效益和竞争力。加大农业基础投入，加强农田水利建设和农业综合开发，高标准农田占比提高4个百分点。加速粮食生产全程机械化，抓好设施农业机械化推广应用，不断提高农业装备水平。提升财政资金支农效益，发挥其杠杆作用，撬动产业、金融等社会资本投入农业。用好国家政策性资金，创新农业保险产品，切实防范农业经营风险。强化农业科技支撑，加强科技人才队伍建设，激活科技创新资源，加快农业科技成果转化应用，提高农业技术推广服务能力，建设农业大数据平台，推进“互联网+现代农业”，着力补齐农业科技创新短板。

构建农业产业体系。按照“4+1”现代农业产业规划，突出主导产业和特色产品培育，大力发展绿色优质农产品。促进产业融合、多元复合，壮大农产品加工业，培育农村电子商务、乡村旅游等新业态，推动农业“接二连三”发展，实施投资超亿元农业产业化项目10个、超3000万元农产品加工项目50个。放大地理标志证明商标市场带动效应，推动农产品标准化生产和质量监管追溯体系建设，加快建设“淮安大米”等一批国家驰名商标农产品示范基地。积极发展稻虾共作、稻绿轮作、农牧结合等种养模式，提高经济、生态、社会综合效益。发挥国家和省级农业科技园区龙头和示范作用，带动设施农业规模化发展，新增高效农业面积6万亩。

培育新型经营主体。壮大农业龙头企业，规范提升农民合作社，扶持家庭农场、专业大户发展，培育社会化服务组织、农村电商等新型主体，新增家庭农场800个、市级以上示范农民合作社100个、省级以上农业龙头企业10个。加大新型职业农民培训力度，鼓励返乡农民工、大学生、退役士兵、农业科技人员投身农村创业，带动农民增收、农业增效。促进农业龙头企业、合作社与农户之间利益联结，扩大联耕联种联管联营试点范围，建设一批产业化示范基地。深入推进农村社区股份合作社和各类专业合作组织建设，发展壮大村级集体经济。

（三）放大特色优势，推进现代服务业转型升级。

聚力项目建设。加强重点项目招引、跟踪服务和动态监测，实施投资超5000万元服务业特色产业项目200个，完成投资630亿元，力争“4+3”服务业特色产业增加值占比达77%。大力发展生产性服务业，加快实施一批市场潜力大、投资效益好、品牌带动力强的优质项目，促进特色服务业向专业化和价值链高端延伸，推动企业降本增效，进一步增创产业发展新优势。

聚焦重点领域。做强现代物流业，重点打造港口、铁路、航空三大核心区，加快建设苏北快递产业园，积极发展集散分拨、仓储配送等产业，努力建设区域性物流枢纽城市。做特健康养老业，促进养老服务与医疗、护理、旅游等产业互动发展，加快培育新型健康养生产业。做精文化旅游业，着力构建“一核两圈五板块”全域旅游发展格局，丰富里运河、西游记文化旅游和洪泽湖、白马湖生态旅游内涵，提升红色旅游影响力，启动乡村旅游民宿建设三年行动计划，努力打造“长三角”旅游目的地城市。做优新兴业态，实施“互联网+”20项行动计划，积极发展数字经济、分享经济、创意经济，加快创建国家电子商务示范城市，着力打造苏北软件和信息服务产业高地。

聚合平台载体。围绕市域空间布局和“4+3”特色产业规划，立足特色优势，引导产业错位发展、专业市场合理布局。推动产业集中集聚，发展提升宁淮现代服务业集聚区、宁淮新兴产业科技园、淮安软件园等载体，打造现代服务业优势产业集群，新认定市级服务业集聚区3-5家，市级以上服务业集聚区入驻企业超过1.4万户，新增投资150亿元，营业收入增长12%以上。

（四）坚持共建共享，推动城乡统筹协调发展。

突出规划引领。坚持精明增长和内涵发展，加快转变城市发展方式，带动空间布局优化和功能提升，促进城乡一体化发展。深化“多规合一”改革，完成城市总体规划修编，加快城市设计、中心城区控规及专项规划修编。统筹布局学校、医院、文体等公共设施，不断完善服务功能，引导人口合理集聚。转变设计理念，抓好建筑形态、绿化景观、街区风貌、地下空间的规划设计，促进各要素之间功能相匹配、形态相适应。加大规划调控力度，坚持项目跟着规划走，强化土地上市的规划管控和市场调节，促进城市有序发展。

增强城市功能。计划投入968亿元，实施793个重点项目，着力提升城市能级。加快功能片区建设，提升淮海中心商业区发展质态，增强水渡口中央商务区集聚能力，加快高铁商务区、空港产业园等建设。不断彰显城市特色，深入挖掘历史文化内涵，启动建设中国漕运城、盐晶堡主题乐园，打造老坝口历史风貌区，实施周恩来纪念地及周边环境整治提升工程，全面应用城市形象识别系统，加快形成城市特色风貌。持续改善宜居环境，整治210个老旧小区，建成6平方公里海绵城市示范区，全面整治易涝片区、道路积水点，实施3座水厂深度处理改造和2座城市污水处理厂提标改造，完成国家水生态文明城市建设试点任务。大力促进互联互通，积极推进连淮扬镇、徐宿淮盐铁路建设和宁淮、沂淮铁路前期工作，开工建设高铁综合客运枢纽；建成503省道等工程，完善国省干线公路网络；加快实施京杭运河淮海南路大桥等项目，打通10个瓶颈路段和拥堵道口；完成流域性防洪、区域治理、河湖水系连通等项目，争取实施淮河入海水道二期工程。

促进统筹联动。以区划调整为契机，优化清江浦区功能布局，增强综合承载力，加快淮洪一体化发展步伐，进一步推进淮安区、淮阴区与主城区融合发展。创新“一县一策”扶持机制，加快盱眙撤县设市步伐，推进涟水副中心、金湖水乡园林城市建设，努力打造各具特色的县域中心城市。突出以城带乡，强化规划引导和产业带动，促进产镇加快融合、资源综合利用、地域风貌彰显，加快建设一批小而美、小而强的特色小镇。全面推广“五位一体”管护机制，提升乡村治理水平，积极推进美丽乡村建设。

创新管理体制。深化城市综合执法改革，推进市容环境法治化管理，实行规划、建设、城管全过程监管，实现一支队伍管执法。建立健全市、区、街道、社区四级管理服务体系，合理调整市和区事权，将管理的关口前移、重心下移。全面建成“智慧淮安”基础数据库和基础网络，实现重点民生领域智慧化应用，完善覆盖市区的数字化城管监督指挥系统。深入推进全国文明城市创建，争取高分通过国家年度测评。

（五）深化改革开放，集聚加快发展新动能。

推进供给侧结构性改革。以提高全要素生产率为目标，有效落实“五大任务”。全面实施去产能工作方案，健全市场化退出机制，严控新增落后产能。积极拓宽去库存渠道，加大住房货币化补贴和棚改货币化安置力度，商品房去化周期降至合理区间，加大调控和监管力度，促进房地产业健康有序发展。加强债务余额限额管理，严控新增债务，加快存量债务化解，稳步推进去杠杆。出台降成本配套措施，切实降低实体经济税费、融资、用能、人工等成本。对照全面小康建设标准，进一步集中有效资源，逐项落实补短板任务。

突破重点领域改革。深入推进行政审批制度改革，积极稳妥开展相对集中行政许可权改革试点，加快建立全市统一的行政权力事项清单，实现权责清单融合，升级完善政务服务“一张网”，加快建设四级一体化政务服务体系，开展盱眙、淮安经济技术开发区综合行政执法改革试点。稳步推进新型城镇化综合改革，开展高沟、车桥等重点中心镇扩权强镇试点，加强农民进城落户政策扶持，探索建立农业转移人口市民化成本分担机制，加快农民工市民化步伐。实施3个功能片区土地利用规划试点，推进同一乡镇村庄优化布局和土地综合整治，不断提升土地节约集约利用水平。深化农村产权制度改革，推进农村土地“三权分置”，拓展土地确权登记颁证成果运用，扩大承包土地经营权抵押贷款试点，依法推进农村土地经营权有序流转。深化金融改革创新，吸引股份制、台资等银行业金融机构在淮落户，积极培育地方金融组织，大力发展普惠金融，引导金融机构向金融中心集聚，加快企业上市步伐。加大国有企业、供销社改革力度，统筹推进企事业单位公务用车、审计机关人财物管理制度等各项改革。

提升对外开放水平。放大台资示范区品牌和省专项支持政策效应，继续实施台资项目倍增、交流平台拓展和101%服务升级计划，建立省级以上开发区与岛内园区合作机制，聚力招引台资产业链高端项目，推动台资产业转型升级。转变招商理念，坚持项目为王、产业为重、效益为本，注重项目的实际投入、税收贡献和产业支撑，形成鲜明的绩效导向。着力提升外资利用质态，新引进总投资超3000万美元先进制造业项目30个以上，注册外资实际到账实现正增长，其中制造业和生产性服务业到账外资增长10%左右、力争占比达50%。突出优进优出导向，加快发展跨境电商等新兴业态，新培育进出口企业100户以上，外贸进出口总额实现正增长。引导企业参与“一带一路”建设，开展国际产能合作。扎实推进国家和省级园区创建，创新体制机制，丰富发展内涵，加速产业集聚，不断提高各类园区对全市开放型经济的贡献率和支撑度。

推进区域合作发展。全面对接“一带一路”、长江经济带、沿海开发等重大发展战略，积极推动淮河生态经济带规划编制工作，支持淮河生态经济带工商联盟开展区域经济合作，推进洪泽湖生态经济区建设和淮河银行、淮河开发总公司筹建前期工作。实质性参与沿海发展联合会运作，共同设立和有效使用江苏沿海发展基金。参与沿海“3+3”港口联动，争取沿东陇海线重大项目布点，拓展与沿海、沿线城市经贸产能合作。实施宁淮一体化发展规划，推动盱眙、金湖、洪泽先行融入江北新区，加快市域整体融入南京都市圈。办好长三角城市经济协调会第17次市长联席会议，不断提升淮安的知名度和美誉度。

（六）坚持绿色发展，增创区域竞争新优势。

扎实开展“两减六治三提升”专项行动。按照省委、省政府的部署，用2-3年时间，以开展“两减六治三提升”专项行动为抓手，有力解决突出问题，有效控制环境风险，尽早实现生态环境质量的根本性好转。详细制订专项行动实施方案和年度工作计划，将所有指标分解到县区、部门，落实到企业、项目，全力打赢生态保护和环境治理攻坚战。着力改善空气质量，大力实施二氧化硫、氮氧化物削减项目，持续开展燃煤锅炉整治、重点企业挥发性有机物和扬尘治理，抓好秸秆机械化还田利用，确保PM2.5浓度降幅、优良天数比例等达到国家和省考核目标。大力防治水污染，坚决取缔不符合环保政策的“十小”生产项目，严格落实河长制，全面实施城市建成区控源截污和黑臭水体整治工程，加强畜禽养殖污染治理，开展水环境区域补偿，确保国、省考核断面达到要求。实施全市土壤污染防治行动计划，启动土壤污染状况调查，开展土壤环境监测。加强化工园区整治，力争通过省专项整治考核。

筑牢生态安全屏障。深化生态文明体制改革，建立资源有偿使用和生态补偿等制度，健全环境治理和生态保护市场体系，加强生态文明绩效评价考核和责任追究，筑牢生态安全机制屏障。坚持总量指标削减、项目环评与规划环评审批联动，加强违规项目清理和违法案件查处，实施环境保护网格化管理，开展敏感目标风险评估，强化突发事件应急响应，筑牢生态安全管控屏障。积极巩固生态创建成果，全面推进国家生态文明先行示范区建设，设立一批生态保护引领区，探索建立碳排放权交易市场，加大城乡绿化造林力度，筑牢生态安全建设屏障。

推动绿色低碳发展。加大传统产业低碳化改造力度，引导企业实行合同能源管理，实施重点节能改造项目30个，节约标准煤6万吨。加快淮安经济技术开发区循环化改造，推进3个市级低碳试点园区建设。发展绿色节能建筑，加强公共建筑能耗限额监管，积极发展装配式建筑，城镇新建民用建筑全面达到绿色建筑星级标准，建成超低能耗被动房示范项目。倡导绿色低碳出行，优先发展公共交通，推广新能源汽车，建设慢行交通示范区，构建绿色交通体系。

（七）加大民生投入，提升公共服务水平。

积极发展社会事业。全面推进教育现代化，完善市区中小学和幼儿园布局规划，建成一批新校区，加大师资建设力度，促进优质资源均衡配置，创建一批国家、省示范性职业学校和职教集团，促进驻淮高校与地方融合发展，恩来干部学院核心区建成投用。扎实推进“健康淮安”建设，深化公立医院改革，加快建立现代医院管理制度，完善基层医疗卫生机构运行机制，实现区域人口健康信息平台高效运转，提升计划生育和妇幼健康服务水平。完善现代公共文化服务体系，稳步推进基层综合性文化服务中心建设，建成市公共数字文化综合服务平台，积极扶持文化精品创作。加强公共体育服务体系建设，大力开展全民健身运动，健全体育后备人才培养体系，办好智力运动、户外运动等国际赛事，促进群众体育、竞技体育和体育产业协调发展。

不断完善保障体系。健全就业保障机制，突出高校毕业生和下岗失业人员两个重点，兜住就业底线，统筹推进困难群体就业。完善扶贫攻坚机制，实现13万低收入人口脱贫、45个经济薄弱村达到“新八有”标准。继续实施全民参保登记计划，进一步扩大社会保险覆盖面，稳步提高退休人员养老待遇、大病保险统筹基金筹资标准、医保报销比例及最高支付限额，完成城镇居民医疗保险和新农合整合，逐步缩小城乡低保差距。完善临时救助、医疗救助和大病保险制度，发挥慈善社会救助补充作用，逐步扩大救助范围。加快建设社会养老服务体系，重视发展老年教育。强化全民国防民防教育，加强后备力量和军人荣誉体系建设，推进军民融合深度发展，争创全国双拥模范城“五连冠”。

全力维护社会稳定。深化平安淮安、法治淮安建设，严密防范和依法打击各类违法犯罪活动，不断提升人民群众的安全感和满意度。推进社会治理创新，促进大数据应用与社会治理深度融合，创新升级立体化、信息化社会治安防控体系，全面深化公安改革，提升社会治理专业化水平。深入开展“七五”普法，增强全民法治观念。推进法治信访建设，不断放大阳光信访升级版效应。开展平安家庭创建，维护妇女儿童合法权益。完善透明食品药品监管体系，积极创建省级食品安全城市。抓好生产、交通、消防、网络、金融等重点领域安全监管，健全应急处置联动机制，提高公共安全保障和突发事件处置能力。进一步落实企业安全生产主体责任，加强执法能力建设，保持安全生产形势稳定可控，努力营造和谐稳定社会环境。

2017年市政府将继续办好民生十件实事，积极支持各县区兴办一批民生实事，进一步改善群众生产生活条件。一是开工建设中心城区45.6公里的内环高架及连接线，提升快速通达能力。二是建设中心城区饮用水备用水源，新建京杭大运河取水口，铺设52.8公里管道，实现长江水与淮河水互为备用。三是在63.5平方公里老城区新改建污水管网和雨水管道，综合治理城市水环境。四是完成1.1万户以上棚户区改造任务，改善3.5万人居住条件。五是新建扩建生态新城高中、淮阴师院附属中学和第一附属小学、市外国语实验小学和20所幼儿园，着力优化教育布局。六是异地新建市妇幼保健院，基本建成市公共卫生中心，不断增强医疗卫生服务能力。七是新投放300辆新能源公交车、3000辆新型公共自行车，方便市民出行。八是提高社会保障水平，城乡居民医保人均财政补助标准由425元提高到470元，城乡低保标准由每人每月520元、400元分别提高到550元、460元。九是促进就业创业，完成城乡劳动者职业技能培训2.6万人次，城镇新增就业6万人，扶持创业1.5万人。十是保障农村饮水安全，巩固提升进村入户集中供水管网，新增受益人口100万人，实现城乡饮用水同源同网同质。

三、全面加强政府自身建设

开启新征程、创造新业绩，要求以更高标准推进政府自身建设。新一届政府将牢记重托、不辱使命，进一步加强现代政府治理理念、治理体系和治理能力建设，全面提高执行力和公信力，努力打造人民满意政府。

倡导务实担当的工作作风。牢记“实干兴邦、空谈误国”的教导，牢牢把握绩效导向，认真负责做好各项工作。以功成不必在我的胸襟，多做打基础、利长远的工作，坚决杜绝“抠着指标凑数字”，确保各项工作经得起历史、实践和人民的检验。强化责任担当，健全领办会办帮办督办推进机制，坚持尽职免责、失职追责，鼓励创新实干，允许探索试错，坚决整治不作为、慢作为、乱作为，确保各项任务一抓到底、务求实效。发扬工匠精神，当好“操盘手”，不断提高科学施政和驾驭经济发展能力。

践行亲商为民的服务理念。加强诚信政府建设，加大政策执行跟踪督查和审计力度，全面兑现服务承诺，坚决惩治政府部门和公务员的失信行为，切实维护群众利益和市场公平秩序。大力发扬“店小二”精神，不断优化101%服务，着力构建“亲”“清”政商关系，营造适宜投资创业的政务服务环境、法治环境、市场环境、人文环境。坚持以人民的根本利益为出发点，认真解决群众关心的热点难点问题，推动政府工作和民生需求无缝对接，让发展更有温度、幸福更有质感。

健全依法高效的运行机制。强化法治思维，依法制规立法，加强行政执法监督检查，提升法治政府建设水平。认真执行议事决策程序，持续深化政务公开，主动接受人大法律监督、工作监督和政协民主监督，不断提升决策科学化、民主化、法治化水平。创新“互联网+政务服务”模式，推进行政权力网上公开、透明、规范运行，扎实开展护航发展专项行动，切实提高行政服务效能。

保持清正廉洁的从政本色。严格遵守政治纪律和政治规矩，认真执行党内法规制度，切实绷紧从严治政这根弦。深化周恩来“五德”教育，大力弘扬廉政文化，全面落实党风廉政建设主体责任和“一岗双责”，注重运用监督执纪“第一种形态”，对苗头性、倾向性问题，做到早提醒、早纠正、早处置。紧盯“关键少数”，严格执行市直单位五项重点权力制约监督规定，坚决惩治各类腐败问题。坚持勤俭办一切事业，压降行政成本，严控“三公”经费，切实把资源和财力用到发展最迫切、群众最需要的地方。

各位代表，新形势催人奋进，新征程任重道远。让我们紧密团结在以习近平同志为核心的党中央周围，在省委、省政府和市委的正确领导下，进一步解放思想、开拓创新、埋头苦干，为实现“两大目标”、加快建设繁荣安康宜居文明的美好淮安而努力奋斗！
